# Narrowing the gap between eye care needs and service provision: the service-training nexus

**DOI:** 10.1186/1478-4491-7-35

**Published:** 2009-04-23

**Authors:** Keith Masnick

**Affiliations:** 1 School of Public Health and Community Medicine, University of New South Wales, Kensington, New South Wales, Australia

## Abstract

**Background:**

The provision of eye care in the developing world has been constrained by the limited number of trained personnel and by professional cultures. The use of personnel with specific but limited training as members of multidisciplinary teams has become increasingly important as health systems seek to extract better value from their investments in personnel. Greater positive action is required to secure more efficient allocation of roles and resources. The supply of professional health workers is a factor of the training system, so it stands to reason that more cost-effective, flexible and available education methods are needed. This paper presents a highly flexible competencies-based multiple entry and exit training system that matches and adapts training to the prevailing population and service needs and demands, while lifting overall standards over time and highlighting the areas of potential benefit.

**Methods:**

Literature surveys and interviews in five continents were carried out. Based on this and the author's own experience, a encies-based multiple entry and exit scheme for eye care in a developing country was derived, modeled and critically reviewed by interested parties in one country.

**Results:**

The scheme was shown to be highly cost-effective and readily adaptable to the anticipated eye care needs of the population. Eye care players in one selected country have commented favourably on the scheme.

**Conclusion:**

The underlying principles used to derive this model can be applied to many eye care systems in many developing countries. The model can be used in other disciplines with similar constructs to eye care.

## Background

### A huge and growing burden

The worldwide number of visually impaired persons, according to current World Health Organization (WHO) definitions is 161 million (best corrected vision ≤ 6/18), of whom 45 million were blind (best corrected vision ≤ 3/60). Of the blind, more than 80% are aged over 50; age-related macular degeneration (AMD) is the most rapidly increasing cause of blindness in this group [1]. Some 75% of blindness occurs in developing countries; Vision 2020 has estimated that 75% of that blindness is preventable [2]. If a legitimate alternative to the WHO standard is used (impairment is defined as presenting acuity of ≤ 6/12 and blindness as presenting acuity of ≤ 6/60) then impairment prevalence will increase by 65% [3]. An Australian study estimated the same change at almost 150% [4]. If those who are refractively blind only for close work were taken into account, this could add at least another 150 million people to the functionally blind [5].

The annual global gross domestic loss (loss of gross domestic product) from blindness and low vision runs into tens of billions of United States dollars [6]. In Australia, about 2% of all health disability-adjusted life years (DALYs) are due to aged-related vision disorders [7]. Unless very substantial improvement is made in the management of visual impairment, increases in the world's aged will impose further burdens of disability and economic loss.

Most eye care is of low to medium complexity, with a very small probability of serious or life-threatening conditions. Provision of corrective lenses and cataract removal account for most of the eye treatments. It has been shown in developing countries that the use of personnel with limited but appropriately specialized training results in high-value outcomes is very cost-effective [8,9]. Unfortunately, the numbers of such personnel are small and training programmes are not presently geared to the expansion of the supply at anything near the rate required to close the gap between need and provision of services.

This paper proposes a public eye care system for developing countries based on a service-staffing paradigm that permits an eye care worker to move both "upwards" and "sideways", reflecting increases in the worker's repertoire of competencies within one vocational stream or crossing over from one vocational stream to another as health demands and occupational preferences change over time. A competencies-based modular training scheme that provides multiple entry and exit points directly matches the needs and structure of the overall service-staffing model and enables personnel to acquire the competencies required to benefit from opportunities for career mobility. This flexible scheme will target deficiencies in eye care delivery with specifically trained personnel, thereby providing a better balance between the opportunity costs of educating eye care personnel for all possibilities and cost-effectiveness of outcomes. The flexibility will reduce issues of oversupply and undersupply and use of personnel. While this model has been developed for Thailand, which has a universal health care system, the principles should be applicable to most developing countries.

### The eye care service-staffing matrix

**Figure 1:**
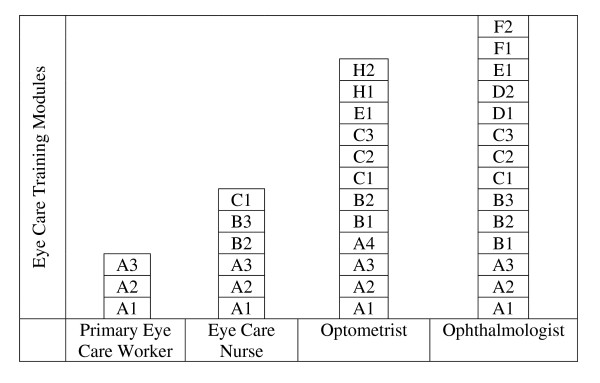
**Traditional modular eye care personnel training**. Note: Although modules in different columns may have the same notation (e.g. C2 = refraction), their content may differ.

Currently in both affluent and less affluent countries, eye care activities – preventive care, screening, diagnosis and treatment – are principally in the hands of expensively trained medical personnel, optometrists and a generally small cadre of eye care nurses/ophthalmic medical assistants. The training and career pathway for such personnel is typically "columnar", as shown in Figure 1, and crossover from one column to another is extremely unlikely.

Throughout the world, government health care systems generally conform to a four- or five-level institutional arrangement, from local clinics to national hospitals. Within these stratified systems, the posts for personnel with more or less exclusive responsibility for eye care can be classified into five vocational groups: (1) eye care clinical officers and medical assistants and nurses; (2) refractionists; (3) ophthalmologists/ophthalmic surgeons; (4) orthoptists; and (5) eye service administrative personnel. Individual personnel can often cover more than one group. In a few countries, a sixth group is composed of cataract surgeons who, although they have completed specialized training in cataract surgery, do not hold a medical degree: they usually have completed formal training as nurses or clinical officers/medical assistants [8].

**Figure 2:**
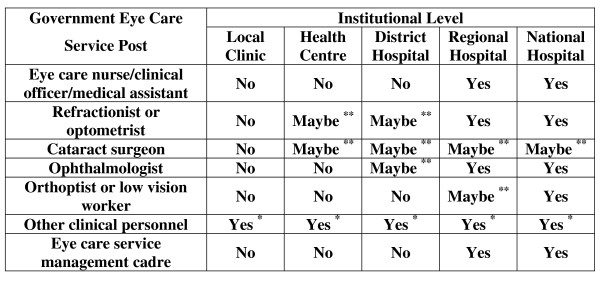
**Basic eye care service staffing matrix**. *Other clinical personnel who perform some primary eye care, e.g. general nurses, general clinical officers/medical assistants or general medical practitioners. **Trained personnel may be available at this level, perhaps on a visiting basis.

**Figure 3:**
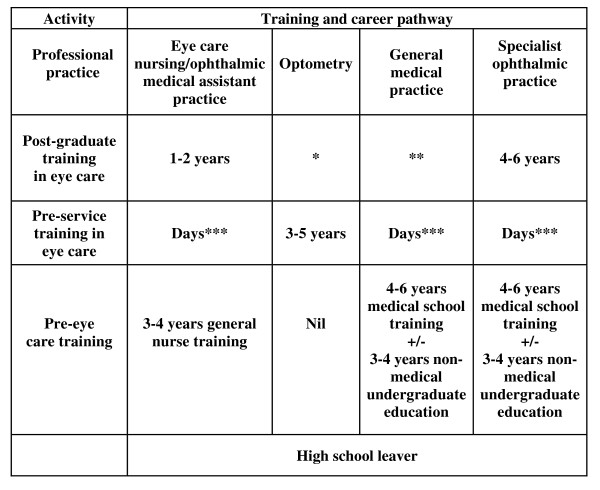
**Training and career pathways, eye care personnel – current career patterns in most countries**. * Small number of places – generally regarded as preparation for a teaching or research career. ** No or very limited formal training in eye care during internship. *** The time spent on eye care-related activities is typically a matter of days rather than weeks, months or years.

This workforce is augmented by general medical practitioners or other staff who perform primary eye care functions at the local clinic or health centre level. In less affluent countries these are typically general nurses and clinical officers/medical assistants who include primary-level eye care within their diverse and wide-ranging skills. Optometrists are, outside the United States of America and the United Kingdom, noticeably absent from the public health system [10]. Figure 2 shows a typical basic eye care service-staffing pattern for a national government eye care service; Figure 3 shows the typical related training.

There are possible variations of this basic paradigm to meet the requirements and resources of particular local and national circumstances. In most countries the government eye care service is augmented by private sector activity. For example, in larger towns and cities a medically qualified ophthalmologist or an optometrist may have established a full-time private practice or combine government employment with some private practice. In urban areas there are usually retail establishments offering eye testing and retailing spectacles and other eye care requisites, perhaps with staff making periodic forays to the surrounding rural areas seeking customers.

### Training the eye care workforce

Underlying the proposed service staffing approach is the principle that eye care can be parceled into a number of discrete functional units, each containing the actions of one or more procedural tasks. For each functional unit a training module must be designed to enable trainees to learn to perform the component function or functions to a specific standard.

All eye care personnel will be required to possess the competencies of some basic functional units, such as elementary visual acuity testing. Other modules will include competencies in more specialized tasks; learning them will be required for employment in particular positions within the service-staffing matrix. For example, an optometrist will need to be competent within the local scope of optometric practice [11] and a cataract surgeon will require competence in certain lens extraction and replacement techniques.

There is nothing new about a modular approach to the training of eye care personnel [12], or indeed to that of many other types of workers. In traditional training programmes, the modules are arranged in columns, each relating to a particular vocational category (see Figure 1) and although some modules may be common to a number of columns, there is no sideways movement between the columns. Even the content of the common modules differs. For example, a refraction course in one module might take one year to complete, while only a few days in another.

In the proposed training arrangements there will still generally be columnar arrangements, and training for a particular vocational group will normally proceed vertically up the training column, but sideways movement from one column to another will be possible. The key to the sideways movement is that the whole training system is based on a universal set of competencies such that each occupation can be defined by its domains of competencies.

Movement between training columns depends on the type and level of training completed before entering specialized eye care training. For example, a person at any point in the eye care nurse training column in Figure 1 could move sideways to an appropriate point in the optometrist column to train as an optometrist, but neither could move into the ophthalmologist column without a bridging course of training to acquire the missing competencies. Despite this limitation, the shift from a strictly vertical vocational training/career approach to a system that permits sideways as well as upward progression provides markedly increased opportunities for career mobility. This mobility facilitates the relatively rapid production of particular categories of personnel currently in short supply and the gradual lifting of overall competence over time.

### Training cataract surgeons

Worldwide, age-related cataracts account for almost half of all non-refractive visual impairment [13]. In Australia, for example, cataract surgery represents approximately 75% of all major eye surgery [14].

**Figure 4:**
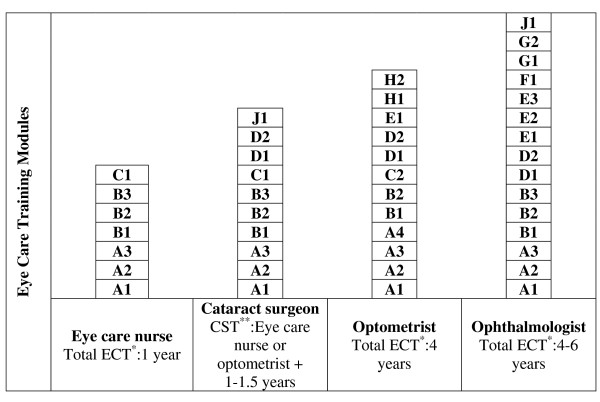
**Example of proposed modular training system for production of cataract surgeons**. * ECT = Eye care training time; ** CST = Cataract surgery training time.

Although cataract surgery is now almost entirely in the hands of medically qualified ophthalmic surgeons, there is ample evidence that appropriately trained personnel can perform procedures within this category as effectively, efficiently and safely as ophthalmologists (Cox, I. (2004). Doctor substitutes in East Africa. African Director CMB International. personal communication) [8,15]. Figure 4 illustrates how the proposed modular training system facilitates the faster production of a cataract surgery cadre than the 10-plus years required for an ophthalmologist. For example an eye care nurse who has completed modules A1 to C1 would move over to commence module D1 on the cataract surgeon training column and acquire the modules Dl, D2 and J1. An optometrist would add the modules Cl and J1. In both cases the total additional training time for qualification as a cataract surgeon would be one to one-and-a-half years.

### The multi-entry/multi-exit (ME/ME) training system

**Figure 5:**
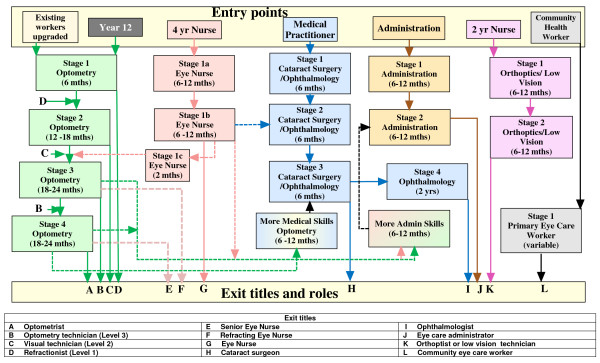
**Training flows in eye care, showing training stage numbers, normal entry and exit points and sideways career movement possibilities (dashed lines)**. "Existing/Upgraded" means the applicant has acquired or must acquire sufficient entry-level education before entering the course. A module's duration (shown in brackets) is a guide only. A full optometry course will take 4–5 years.

The modular structure of the training system permits a range of choices as to entry, exit and then re-entry and exit within and between vocational training streams. Figure 5 shows in more detail how individuals entering the service-staffing matrix with different types and levels of prior experience may navigate their way through the matrix to reach posts that are appropriate to their developing interests and competence. For example, a high-school leaver who enters the training system and completes the stage 1 optometry module may enter the workforce as an entry level refractionist. She or he may subsequently decide to return to the training system to select and complete further modules that would qualify her or him for higher-level posts within the refractionist/optometrist stream, or for posts in other streams within the matrix, such as cataract surgery. Alternatively, on re-entering training she or he may prepare for a career in eye care service administration, for example. This figure demonstrates some common paths that may be taken by people of differing pre-entry status.

Of particular note in this model is that the short training time for stage 1 optometry is designed to equip people to perform at an acceptable level of refraction, optical mechanical skills and modest non-refractive detection. The short course:

• could be taught either intensively or part-time and can include a distance learning component;

• will quickly supply a large number of relatively trained personnel to refract, make spectacles and detect eye conditions at a basic level;

• will appeal to existing eye care workers, most of whom have not graduated from year 12, by easing them back into education;

• will be reasonably inexpensive and highly cost-effective;

• will produce graduates who are more likely, given the restricted training, to have modest ambitions and to stay locally;

• will introduce a novel degree of specialized eye care at the lower end of health care in a way that is acceptable and affordable for the local community.

Optometry stages 2 to 4 are intended to supply personnel to the provincial and higher levels of the health service. Because the multi-entry/multi-exit training system is coupled to the service staffing matrix, the rate and direction of an individual's movement within the matrix is to some degree a matter of personal choice. However, there are restrictions. One is the availability of the modules in terms of time and place. Second, although a training place may become available, a person's decision to leave his or her current employment to undertake further training is necessarily shaped by practical considerations. The third restriction is the availability of employment opportunities within either the government or other service sectors upon completion of a training course. The possible movement back-and-forth between working in the eye care service and spending time within the training system raises questions as to who provides income to the employee-turned-trainee and who meets the cost of training.

### Competence and competencies-based training

While the notion of modular training is relatively straightforward and easily understood, there is a growing literature revolving around and sometimes obfuscating the meaning of such terms as competence, the competent practitioner, the competent manager, the competencies-based curriculum and competencies-based training.

**Figure 6:**
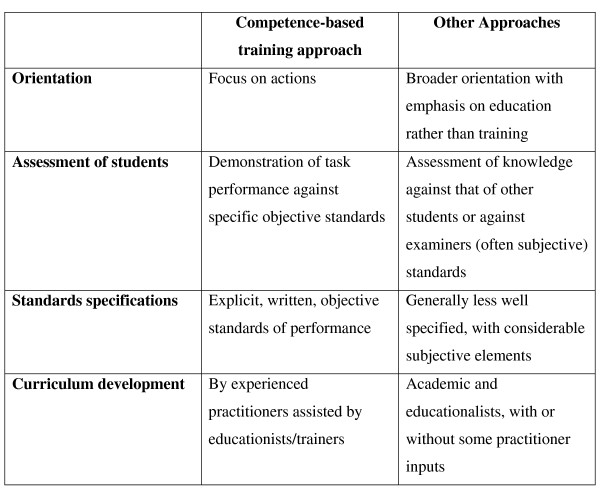
**The competence-based training approach compared with some other approaches**.

In the context of the eye care service and training approach proposed in this paper, competence – or to be more precise, a set of entry level competencies – is essentially gained from the teaching and learning of the knowledge, skills and behaviour required to perform a specified task or set of tasks at specified standards of effectiveness and efficiency. Figure 6 compares the competencies-based training approach espoused in this proposed plan to other approaches.

A commonly voiced criticism of the competencies approach is that the performance of complex tasks requires the interaction of a significant number of elements that cannot be reduced to objective assessment [16]. Thus some authors have suggested that medical diagnostics is an art and not a teachable science and that there may be many ways to the final conclusion [17,18]. Since the concern here is the urgent expansion and staffing of a service that is currently grossly understaffed in countries where training and other relevant resources are in short supply, the competencies-based training approach delineated in Figure 6 is both appropriate and justifiable.

### Implications of implementation

Improvement in services rarely comes without some costs. Some of the major implications of implementing the proposed plan are reviewed here, starting with some of the financial implications.

#### Eye service-related costs – and benefits

Overall eye care service and training costs will inevitably increase as the volume of activities is increased. However, because of the greater efficiency of the service delivery resulting from changes in workforce mix and productivity, the increase will be less than would be achieved by simply expanding present service and training arrangements with no change in efficiency. How the additional costs will be met and the equity implications of the financing arrangements are matters beyond the scope of this paper. The additional expenditure has to be weighed against reduction in the huge and increasing gross national losses attributable to visual impairment.

#### The community at large

With expansion of preventive and early detection activities, one may anticipate some slowing down in the rates of incidence and progression of visual impairment in the population, although with the current backlog of unmet need and future demographic changes, the impact of these primary level activities will not be immediately apparent. Despite the extra costs of the improved services, rewards will manifest themselves in such terms as increased productivity, communal and individual well-being and reduced care resources.

#### Eye care delivery system structure and management

The relative public-private provision contributions vary very widely among countries [19]. The structure of government eye services within countries is generally hierarchical. Private services follow a pattern of private ophthalmology and private manufacture, import/export, wholesale/retail distribution of spectacles and other eye care appliances and equipment. It has to be said that the multi-entry/multi-exit arrangements proposed do require greater managerial and administrative activity. This has to be weighed against the benefits derived from increased staff motivation and performance.

#### The eye care workforce

Expansion of the eye care workforce is likely to occur in both the public and private sectors of eye care provision, but more particularly in the publicly-funded government sector. The mix of personnel within the eye care services will change, with relatively larger increases in the numbers of eye care practitioners such as optometrists and non-medical cataract surgeons. The work pattern and productivity of the more skilled will be more specialized as the less complex case management becomes the responsibility of other appropriately trained personnel. Service expansion and the adoption of the proposed training and staffing offer wider training and employment opportunities and open up greater career path flexibility to personnel entering or already employed within the eye care delivery system.

#### The eye care personnel training system

Staffing the expanded services will entail increased commitment of resources to the training of personnel. Some adjustment in course structure and content will be required to implement the competencies-based modular training. The performance standards on which modules are widely founded and a universal compendium of training syllabi, methods and materials can be developed and shared among training programmes internationally. Additional training personnel will be required, as will teacher training programmes oriented to the use of the methods and materials of the modular programmes. Practising personnel will be required to play a larger role in training and in mentoring graduates from training programmes.

### Prospects for adoption and successful implementation of the proposed plan

Basic eye care is relatively low in direct cost; intervention is relatively simple and has generally a very favorable outcome. Recognizing the current and growing gap between the need for eye care and the provision of services and acknowledging the heavy economic burden of visual impairment, one has to ask why in virtually all countries eye care services rank relatively low on government lists of health care priorities.

As with so many similar questions relating to health service provision, part of the answer is that obtaining eye care is not a great personal problem for relatively affluent people who make or influence health-related political decision-making, nor is other people's visual impairment thought of as a potential threat to their own well-being.

One has to say that this plan, because it promotes more efficient use of resources, may gain some acceptance in situations where government decision-makers are thinking of "doing something about improving eye care services" such as is occurring in Thailand, where the model presented here is being favourably evaluated by both government and the existing ophthalmic community. Interprofessional disputes should not be allowed to constrain the supply of services; the use of common competencies and training should reduce such conflicts and increase interprofessional respect. The difference between demand and supply is so large in most developing countries that with everyone playing his or her part effectively, there will more than enough rewards for all. However, it would be easy to perpetuate a laissez-faire attitude rather than commit the very considerable additional resources required to significantly narrow the gap.

The proposals presented here are adaptable to existing eye care delivery systems (and even other similar health fields) in probably all countries in the world, but are more likely to be adopted in less affluent countries. The cost of failure to narrow the gap will be measurable in terms of increased gross domestic loss and the ever-increasing personal burden of visual impairment.

## Competing interests

The author declares that they have no competing interests.
